# The expression of p63 is associated with the differential stage in nasopharyngeal carcinoma and EBV infection

**DOI:** 10.1186/1479-5876-4-23

**Published:** 2006-05-29

**Authors:** Can Guo, Zhi-Gang Pan, Da-Jiang Li, Jing-Ping Yun, Mei-Zhen Zheng, Zhe-Yu Hu, Li-Zhen Cheng, Yi-Xin Zeng

**Affiliations:** 1 State Key Laboratory of Oncology in Southern China; 2 Department of Experimental Research, Sun Yat-sen University Cancer Center, 651 Dongfeng Road East, Guangzhou 510060, China; 3 Department of Pathology, Sun Yat-sen University Cancer Center, 651 Dongfeng Road East, Guangzhou 510060, China

## Abstract

**Background:**

Nasopharyngeal carcinoma (NPC) is common among Southern Chinese and the main histology is the undifferentiated carcinoma associated with Epstein-Barr virus (EBV) infection. p63 is a recently proved member of the p53 family based on the structural similarity to p53, but its function in NPC is still unknown. This study was aimed to investigate the association between p63 and NPC.

**Results:**

p63 was expressed in 100%(202/202) of nasopharyngeal carcinoma (NPC) tissues but not in 29 nasopharynx inflammation and 17 non-cancerous nasopharyngeal epidermises on a tissue microarray by immunohistostaining. Further investigation suggested that the p63 expression was associated with the differential stage of NPC: p63 strong staining in Keratinizing squamous cell carcinoma, differentiated non-keratinizing NPC and undifferentiated non-keratinizing NPC presented the percentage of 5/8 (62.5%), 43/48 (92.5%) and 50/50 (100%), respectively. A significant difference (*p *= 0.001) existed between the keratinizing and non-keratinizing groups. No pathogenic mutations were detected in p63 gene in 12 primary NPC tissues and matched peripheral blood lymphocytes (PBL). Half-life measurement study revealed distinct stability of p63 protein in the different cell lines, especially between the carcinoma cell lines with EBV infection and the non-cancerous cell lines. The results of immunoprecipitation suggested a direct interaction between Epstein-Barr virus nuclear antigen 5 (EBNA-5) and p63 protein in NPC, and this binding would increase the stability of p63.

**Conclusion:**

Our data suggested p63 might be used as an adjunct diagnostic marker of NPC and contributed a new way to understand the contribution of the EBV in the pathogenesis of NPC.

## Background

Nasopharyngeal carcinoma (NPC) is an epithelial cancer, the histology of which ranges from well-differentiated, keratinizing squamous cell carcinoma to undifferentiated, non-keratinizing carcinoma according to the World Health Organization (WHO) histological classification of the tumors. The etiology of NPC is thought to be associated with genetic susceptibility, Epstein-Barr virus (EBV) infection and some other environmental factors [1-5]. NPC has remarkable geographic and population differences in incidence. In the high incidence area – Southern China, 90% of the NPC patients are undifferentiated, and virtually all nonkerotinizing undifferentiated NPC are associated with EBV [1,6,7].

The tumor suppressor gene p53 is mutated in more than 50% of human cancers [8] and 69% of human head and neck squamous cell carcinomas(HNSCC) [9]; but it was rarely mutate in NPC [10-13]. p63 was identified on the basis of the structural similarity to the p53 tumor suppressor protein. Differing from p53, the p63 gene generates two major types of protein isoforms – TAp63 and ΔNp63 because of two promoters. TA isotypes contain a transactivation domain homologous to that of p53 and can mimic p53 transactivation function, whereas ΔN isotypes lack this domain and block reporter activity mediated by p53 and TA-p63 [14-16]. p63 is essential for regenerative proliferation in limb, craniofacial and epithelial development [17,18]. Over-expression of p63 was observed in many human cancers, especially these less differentiated tumors [19-24].

Epstein-Barr virus (EBV) is a ubiquitous human herpes-virus associated with the development of both lymphoid and epithelial tumors. [25-27]. Initial studies suggested that it was involved in pathogenesis of NPC [2,4,28-30]. The Epstein-Barr virus nuclear antigen 5 (EBNA-5), highly spliced mRNA from the major IR1 (BamHI-W) repeat region of the virus genome, is proved to bind to p53 protein in Burk's lymphocyte [31,32]. The complex of the two proteins can increase the accumulation of the p53 in the nuclear.

Although p63 has high structural similarity according to p53, they do not share similar function. The role of p63 in NPC is still unclear. It was observed in this study that p63 was highly expressed in NPC and associated with the differential stage of NPC. p63 gene took no mutation in NPC, and nevertheless the stability of p63 protein in the carcinoma cell lines was much higher than that in the non-cancerous cell lines. The results of immunoprecipitation demonstrated that p63 could interact with EBNA5 *in vivo*, suggesting an important role in the EBV infection and NPC development.

## Materials and methods

### Immunohistochemical assay

All tissue samples were obtained from the Sun Yat-sen University Cancer center from 2000 to 2004. Immunohistochemistry was performed on 202 undifferentiated NPC samples, 29 nasopharynx inflammation and 17 non-cancerous nasopharyngeal epidermises on a tissue microarray. Other 106 NPC tissue samples of different histologic subtypes were used for further investigation. All the tissue sections were stained with H&E and microscopically examined to ensure the reliability of the diagnosis. Sections were deparaffinized and rehydrated through xylene and alcohol. After incubated with 3% hydrogen peroxide for 30 min at room temperature to block endogenous peroxidase, these sections were then rinsed in 0.01 M sodium citrate buffer (pH 6.0) for 15 min at 95°C to unmask tissue antigens. Immunostaining was performed with p63 4A4 (Santa Cruz) at 4°C overnight. The sections were then developed according to the manufacturer's recommendations (PV-9000 Polymer Detection System, Golden Bridge International) and counterstained with haematoxylin. The evaluation of p63 expression was assessed semiquantitatively by staining intensity as negative, weak or strongly positive according to Carcangiu's method [33]. These data were all assembled in double blind fashion by two independent investigators without knowing the patients' clinic pathological features and follow-up data.

### Cell culture

C666-1 (a nasopharyngeal carcinoma cell line with EBV infection), CNE-1 (a highly differentiated nasopharyngeal carcinoma cell line), CNE-2 (a poorly differentiated nasopharyngeal carcinoma cell line), HEK293 (a human embryonic kidney cell line) and B95-8 (an EBV-positive Burkitt's lymphoma cell line) were maintained in PRMI 1640 (GIBCO) supplemented with 10% fetal bovine serum (Hyclone). NP69 (a nasopharyngeal epithelial cells) was cultured by Keratinocyte-SFM (GIBCO) with Bovine Pituitary Extract and rEGF. Cells were incubated with 5% CO_2 _at 37°C.

### PCR and DNA sequencing

Twelve primary NPC tissues and matched peripheral blood lymphocytes (PBL) samples were used for p63 mutation analysis. The PCR products from NPC tissues were firstly purified using the PCR purification Kit (Qiagen) and A-tailed. Then the A-tailed PCR products were ligated into pGEM-T Easy vector (Promega) and propagated in *E*.*coli *strain DH5α. At least 10 recombinant positive clones of each sample were sequenced. The PCR products from matched PBL DNA were subjected to direct sequencing. Both the sequencing results from NPC tissue and PBL were analyzed by aligning to the p63 sequence (Genebank:AB010153).

### Measurement of p63 half-life

Cycloheximide (CHX) was added at 100 ug/ml and total protein was isolated at 0, 1, 2, 4, 8, 16, 24 and 32 hour after drug addition. The expression of p63 was quantitatively determined by Western blot and normalized by β-actin abundance. The half-life was calculated numerically by using FORECAST (Excel).

### Immunoprecipitation

Cells (3 × 10^7^) were washed twice with ice-cold PBS and dissolved in 200 ul of lysis buffer (20 mM HEPES pH 7.9, 100 mM NaCl, 1% NP-40, 0.5 mM EDTA, 0.5 mM PMSF). Disrupted the cell lysate by sonication (10s on ice), spun down cell extracts at 12000 rpm for 10 minutes at 4°C. The immunoprecipitation were then performed according to the Catch and Release v2.0 Kit (Catch and Release v2.0, *UPSTATE*) recommendations. Lysates were incubated with the anti-EBNA5 monoclonal antibody JF186 (a gift from Karolinska Institute) at 4°C overnight on a rotator by using a Catch and Release spin columns. Washed the Column 3 times with 400 μl of 1X Wash Buffer, spinning at 5000 rpm 15–30 seconds for each wash. Protein bound to the beads was eluted by Denaturing Elution Buffer containing 0.5% β-mercaptoethanol. Proteins were separated by 10% SDS-PAGE, and then transferred to a PVDF membrane (ROCHE). The membrane was incubated with p63 4A4 (Santa Cruz) at a 1:500 dilution. To further identify this result, we also precipitated cell lysates with p634A4 and then did Western Blot with JF186. Western Blot was done by using the Phototope-HRP Western Blot Detection System kit according to the manufacturer's instructions (Cell Signaling).

### Statistical analysis

Data analysis was performed by using SPSS12.0 software package. Pearson Chi-Square Test was used to test the association between the p63 protein expression and the differential stage of NPC. *P *< 0.05 was considered to be statistically significant.

## Results

### Highly expression of p63 in NPC

**Figure 1:**
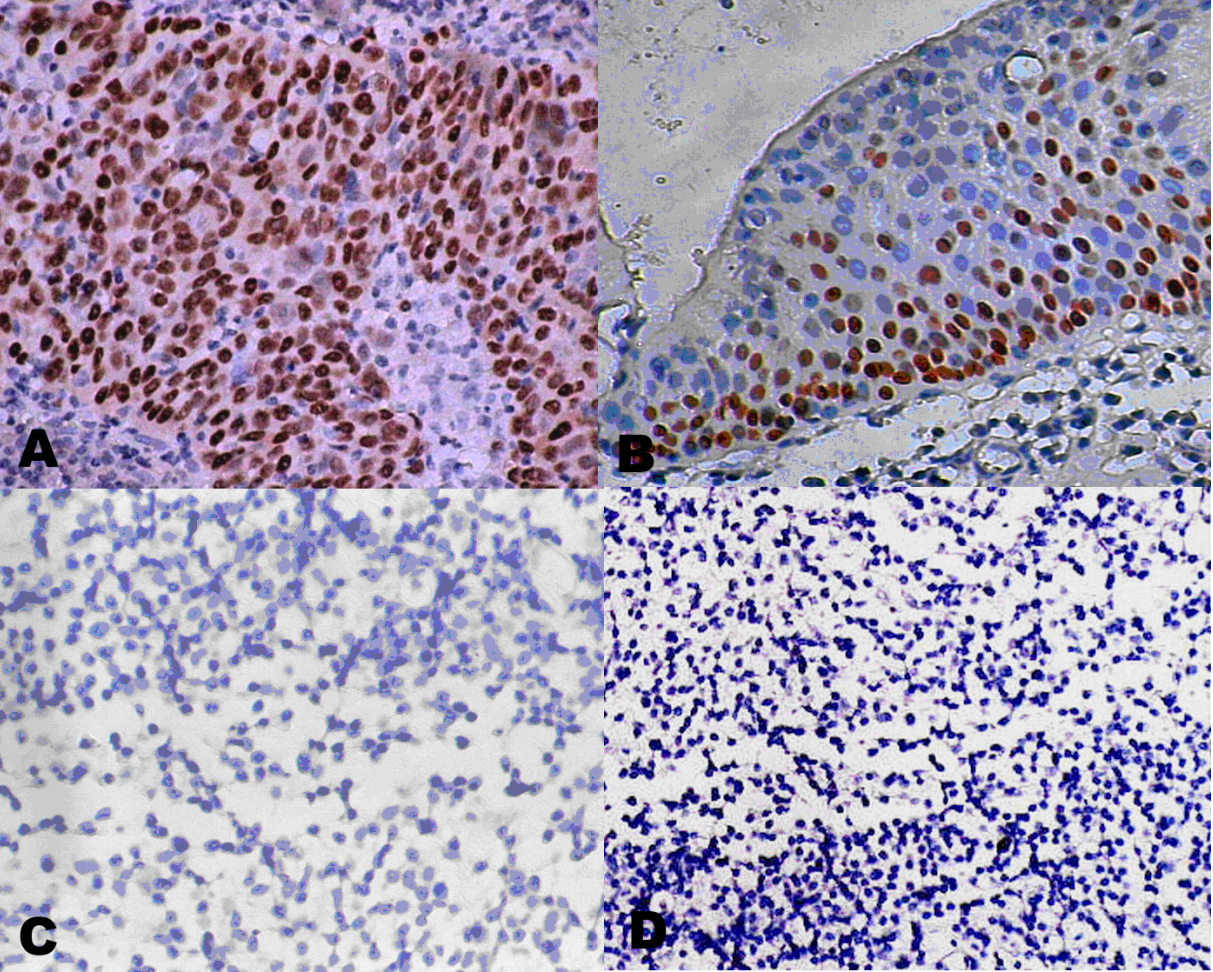
**Immunohistochemical demonstration of p63 protein expression**. Strong positive staining of p63 protein in NPC(A) and the basal layer cells with proliferate potential(B); negative staining of p63 protein in nasopharynx inflammation (C) and non-cancerous nasopharyngeal epidermises(D). (All the photomicrographs were taken in high-powered, ×400).

All 202 undifferentiated NPC samples showed strong positive staining (Fig 1A), but 29 nasopharynx inflammation (Fig 1C) and 17 non-cancerous nasopharyngeal epidermises (Fig 1D) were negative staining. The different expression patterns of p63 staining in the tissue chip were described in table 1. We also found p63 was highly expressed in the cells with proliferative potential (Fig 1B) and the shade of staining was correlated to cell differentiation potency. In addition, recent studies revealed that p63 is highly expressed in the basal and suprabasal epithelium [34]. Totally, our data demonstrated the expression pattern of p63 was remarkably different between non-cancerous nasopharyngeal and NPC and revealed that p63 could be used as a valuable diagnostic marker in clinical adjuvant diagnosis of NPC.

**Table 1:** Expression of p63 immunohistochemical staining in the tissue chip

Nasopharyngeal carcinoma	n = 202	strong positive staining
Nasopharynx inflammation	n = 29	negative staining
Non-cancerous nasopharyngeal epidermis	n = 17	negative staining

### The expression of p63 is associated with the differential stage of NPC

**Figure 2:**
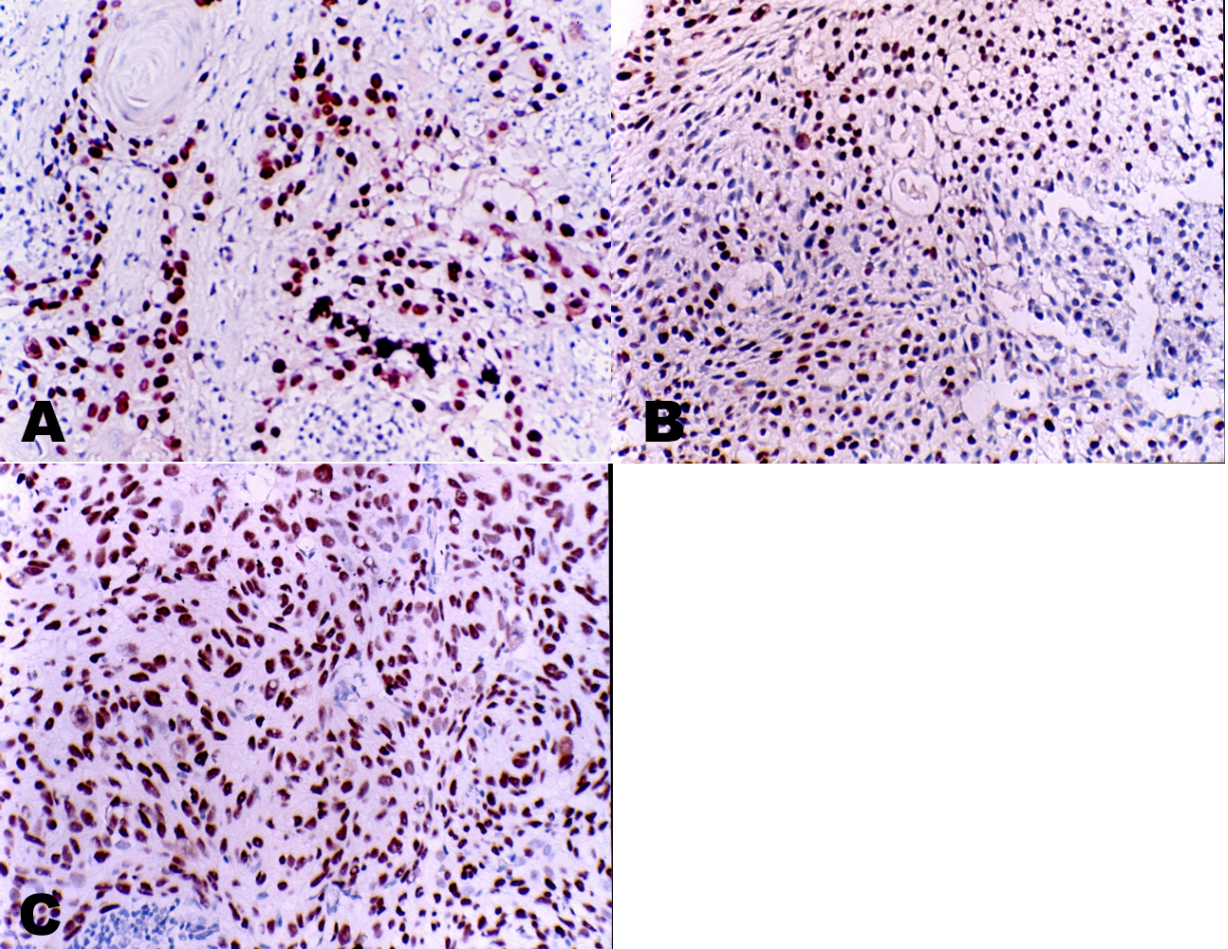
**Immunohistochemical demonstration of p63 protein expression in different histology NPC**. Strong nuclear staining of p63 in keratinizing NPC (A) differentiated non-keratinizing NPC (B) and undifferentiated non-keratinizing NPC (C)(All the photomicrographs were taken in high-powered, ×400). The staining intensity is associated with the differential stage of NPC.

To further investigate the relationship between the p63 expression and the differential stage of NPC, we analyzed 106 NPC tissue samples of different histological subtypes, containing 50 undifferentiated carcinomas, 48 differentiated carcinomas and 8 keratinizing squamous cell carcinomas. The different expression patterns of p63 in different differential stage NPC were displayed in figure 2. All the tissue samples showed nucleus staining, while the intensity of the staining was different with the degree of malignant (Table 2). A significant difference (*p *= 0.001) existed between the keratinizing and non-keratinizing groups. As the keratinizing squamous cell carcinomas was very rarely in the NPC patients of Southern China, we only collected 8 samples in Sun Yat-sen University Cancer center from 2000 to 2004. Although the sample number was few, it indicated a tendency of weak p63 expression in the Keratinizing squamous cell carcinoma. Our data suggested the expression of p63 protein was associated with the differential stage of NPC.

**Table 2:** Expression pattern of p63 in 106 NPC samples with different differentiation status

Differentiated status		Weak staining	Strong staining
Keratinizing squamous cell carcinoma	n = 8	3 (37.5%)	5 (62.5%)
Differentiated non-keratinizing NPC	n = 48	5 (7.5%)	43 (92.5%)
Undifferentiated non-keratinizing NPC	n = 50	0 (0%)	50 (100%)

(*x*^2 ^= 14.934, df = 2, *p *= 0.001)

### Mutation analysis of p63 in NPC

Fourteen exons of *p63 *were amplified and examined in 12 primary NPC tissues and matched PBL samples. Since NPC tissues are confounding with large number of infiltrating lymphocytes, direct sequencing of PCR products may not reveal the real mutation status. We used the PCR-cloning sequencing strategy to ensure the purity of the carcinoma cells. Our study indicated the same results to other studies. Unlike p53, p63 mutation was uncommonly found in human cancer cell lines and tumors [35-37], although it was strongly associated with the development abnormality diseases [17,18,38]. The results suggested that p63 gene mutation was not responsible for the high expression of p63 protein.

### The expression of p63 is associated with its stability

**Figure 3:**
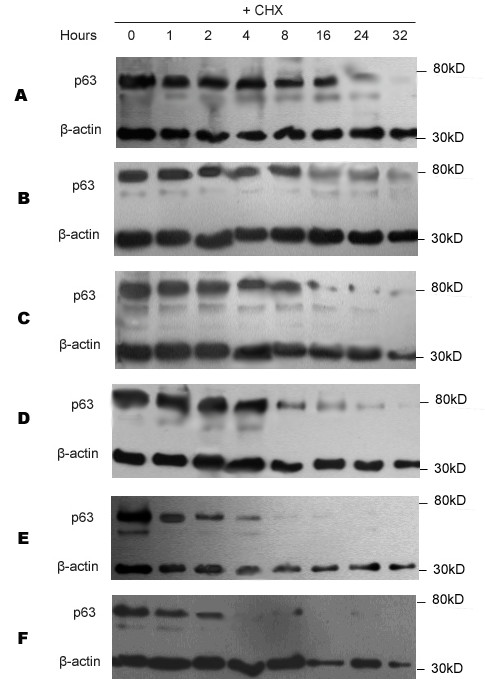
**Stability of p63 with CHX treatment**. CHX was added at 100 ug/ml and total protein was extracted as indicated. Western blots were performed with p63 and β-actin in (A) B95-8 cells, (B) C666 cells, (C) CNE2 cells, (D) CNE1 cells, (E) NP69 cells, (F) 293 cells.

**Figure 4:**
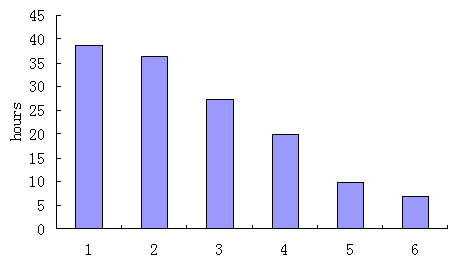
**Half-life of p63 in the different cell lines**. The half life of p63 was 38.64h, 36.42h, 27.47h, 19.74h, 9.76h, 6.93h accordingly to B95-8cells, C666 cells, CNE2 cells, CNE1 cells, NP69 cells and 293 cells respectively.

Stability of p63 in the different cell lines was shown in Figure 3. A time course using cycloheximide at 100 ug/ml was used to obtain an accurate determination of the half-life of p63 and demonstrated differences in the degradation of p63 in different cell lines. The expression of p63 was quantitatively determined by Western blot and normalized by β-actin abundance. The half-life of p63 in different cell lines was described in Figure 4. The interaction of p53 with other proteins is known to alter the stability of p53. In this study, we found the half-life of p63 in the carcinoma cell lines was clearly longer than non-carcinoma cell lines, especially the carcinoma cell lines with EBV infection.

### Direct interactions between p63 and EBNA-5

To further identity the molecular mechanism of high expression of p63, we performed co-immunoprecipitation with p63 and EBNA5. The protein complex of p63 and EBNA-5 were tested in both the C666 cell line and B95-8 cell line, and NP69 was used as a negative control (Fig 3). Like p53, p63 was rarely mutated but take high expression in NPC. High expression of p53 was indicated in inactive form due to the binding with EBNA5 in Burk's lymphocyte [31,32], and the complex of the two proteins led to increased accumulation of p53 in nuclei. p63 have a high amino acid identity to p53. Our study also revealed that the interaction between p63 and EBNA5 might contribute for the stability of p63.

## Discussion

Despite structural and functional similarities between p63 and p53, there are many differences. Inactivation of wild type p53 is indicated in most human cancers due to mutation or interaction of other protein. Previous studies demonstrated that some viral proteins could bind to p53, to prevent its degradation and accumulate in the cell nuclear, and results in high expression. In this study, we found that p63 is highly expressed in NPC, especially the histology of undifferentiated. Considering no mutation detected in NPC, we concluded that gene mutation was not responsible for the high expression of p63 in NPC. p53 protein is labile and its activity is regulated by its degradation and its level of stability. p53 levels are controlled largely by Mdm2. Mdm2 can also bind with p63, but Mdm2 are not able to repress p63-induced transcription or to affect its half-life [39]. In addition, other researchers found that Mdm2 helped increase transcriptional activity and the protein level of p63 [40]. The regulation of the stability of p63 is complex and still poorly understood. Our study suggested a distinct difference of half-life between the carcinoma cell lines with EBV infection and the non-cancerous cell lines. And we found that there is a directly interaction between the EBNA-5 and the p63 protein in NPC by taking the co-immunopreciatiate experiment. These results suggested the interaction with the virally encoded protein might contribute to increase the stability of p63. Although the expression pattern of the p63 is researched generally, the mechanism of its high expression remains an enigma. Therefore, our research was providing a novel mechanism for the degradation of p63.

The role of p63 in tumorigenesis is complex. p53 was firstly heralded as an oncogene because of its potent transformation capabilities and its robust expression in human tumors. However, it was later discovered that only mutant p53 was oncogenic, and that wild type p53 was a tumor suppress gene. Differing from p53, p63 was rarely mutated or inactivated in human cancers, and p63 null mice failed to develop tumors [17,18,37]. Our results showed that the p63 protein was highly expressed in NPC and associated with its differential stage, suggesting it to be a valuable diagnostic marker. In South China, the area with high incidence of NPC, 90% of the NPC patients are undifferentiated, and nonkerotinizing undifferentiated NPC are all associated with EBV [1,6,7]. NPC is unique in that the exogenous EBV is present exclusively in the cancer cells, but not in the surrounding normal tissues, providing an obvious exploitable opportunity [41]. The expression of p63 takes a completely identity of the presentation of EBV. p63 is highly expressed in the carcinoma cells but not in the normal tissues. This is strong evidence to demonstrate the association between the p63 and EBV.

EBV plays an etiological role in the development of NPC. However, how EBV contributes to NPC formation has not been clearly elucidated. Our results proved that the interaction of p63 and EBNA5 *in vivo*. EBNA-5 was required for B-cell transformation [42] and efficient outgrowth of lymphoblastoid cell lines (LCLs) [43]. Previous studies suggested that it was mainly co-operated with EBNA-2 [44]. EBNA2 can transactivating the latent membrane protein 1 of EBV (LMP1) [45]. Numerous studies have revealed the oncogenic properties of LMP1, including increasing cell proliferation and invasion; inhibiting apoptosis, senescence and differentiation [46-49]. LMP1 is conformed as EBV oncogene in NPC [50,51]. We think the p63 may impose an impact on LMP1 by interaction with EBNA2. But how they exert the effect and what are the detailed mechanisms making them to function are still waiting to be discovered.

Totally, our study identified a high expression of p63 in NPC cells and the level of p63 expression was correlated with the differential stage of NPC, suggested p63 might be used as an adjunct diagnostic marker of NPC. And the interaction between p63 and EBNA5 might contribute for the stability of p63. These results implied an important role of p63 gene in development of NPC and prompted a new way to understand the contribution of the EBV in the pathogenesis of NPC.

## Authors' contributions

YXZ and GC were responsible for study design. CG performed the experiments and drafted the manuscript. ZGP and DJL contributed to collection of specimens and mutation analysis. JPY helped in the immunohistochemical assay. MZZ and YZH participated in the data analysis and western-blot. LZC helped in the cell culture. All authors read and approved the final manuscript.

**Figure 5:**
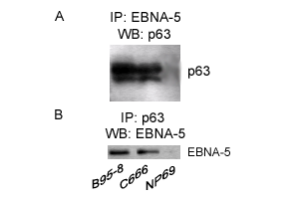
**Co-immunoprecipitation of p63 and EBNA-5**. (A) Cell extract were precipitated with the JF186 and analysed by Western blot with p63. (B) Cell extract were precipitated with the p63 and analysed by Western blot with JF186.
